# Clinical Usage and Economic Effectiveness of a Recently Developed Epidermal Autograft Harvesting System in 13 Chronic Wound Patients in a University-Based Wound Center

**DOI:** 10.7759/cureus.878

**Published:** 2016-11-14

**Authors:** Angela Hulsey, Paul Linneman, Jeff Litt

**Affiliations:** 1 Medical Student, University of Missouri, Columbia, Missouri; 2 Surgery ICU, University of Missouri, Columbia, Missouri; 3 Division of Acute Care Surgery, University of Missouri, Columbia, Missouri

**Keywords:** epidermal autograft, chronic wound, epidermal blister, diabetic ulcer, venous ulcer, wound healing, lymphedema wound

## Abstract

Introduction: Chronic wounds are a significant healthcare problem in the United States. Their costs approach 25 billion dollars in the United States. Current wound-care treatments of local wound care, moist dressings, and source control, while necessary for wound healing, are frequently not enough to ensure complete wound closure. The current surgical technique of split-thickness skin grafting is an operative procedure, painful, time-consuming, and leaves significant donor site wounds. A recently developed and marketed epidermal autograft harvester was tested at our university hospital wound center on 13 patients with wounds of various etiologies. Their clinical outcomes were evaluated, as were the costs associated with its usage compared with the potential costs of continued wound care without autograft placement.

Methods: Thirteen patients whose wounds appeared to have "stalled" or reached a plateau in healing by measurement data and visual evidence were chosen to receive an epidermal autograft to accelerate wound closure. Wound-types included diabetic ulcers, venous or lymphedema-related ulcers, surgical site wounds, and traumatic wounds. Time-to-healing in days, when applicable, was captured. Wound center billing and charges were available and evaluated for nine of the 13 patients. Costs of standard care continuation compared with the cost of epidermal autograft technology usage were compared.

Results: Healing rates were 62%; eight of the 13 patients had healed within four months, two were lost to follow-up, and three have wounds that remain open. Four of the patients healed in less than one month. The comparatively rapid closure of the open wound(s) post-epidermal autograft placement potentially reduced healthcare costs based on charges at an average of $1,153 per patient and yielded an average of $650 to the wound center, not applying the routine costs of dressings applied in the center.

Conclusion: The epidermal autograft harvester accelerated healing in eight of the 13 of the patients (62%) we treated at the time of the writing of this article. By accelerating wound healing in our patient population, costs associated with subsequent wound care seem to have decreased to a dramatic degree and wound center finances have improved. No wound recurrence has been noted once the wounds had healed in our year-long experience with the technology. In addition, the procedure has been well-tolerated and easy to perform. Given the improved outcomes, cost-savings, and a better financial outlook for the wound center, utilization of the novel epidermal autograft harvester is proving itself to be in the “win-win” category of wound care treatments.

## Introduction

Chronic wounds are sometimes described as a “silent epidemic” in the American health system. Often accompanied by co-morbid conditions, they pose a major threat to public health and are a major financial burden on the United States (US) economy. In 2009, it was estimated that 6.5 million patients developed chronic wounds in the US, with an estimated cost exceeding $25 billion dollars spent on their treatment and care [1]. Pressure ulcers are regrettably becoming too common in an increasing number of vulnerable patients – the bedridden, immobile, and/or insensate. Patients 65 years or older accounted for 72% of all of the hospitalized patients reported as having developed a pressure-related wound, with 90% of those patients insured by government health programs [1]. Expenditures on treating pressure ulcers are estimated to exceed $11 billion per year [1]. Similarly, a reasonable estimate is that up to 35% of all diabetics will develop a diabetic/neuropathic foot ulcer over the course of their lifetime [1]. In 2007, the estimated treatment cost of each foot ulcer was between $7,439 and $20,622, with an estimated $9 billion spent on diabetic foot ulcer care in 2001 [1]. Many of these diabetic patients develop multiple and/or recurrent ulcers over their lifetime and eventual amputations are shockingly common. This cost estimate does not include the hidden or indirect costs associated with loss of productivity, the emotional toll on patients and their families, and the resultant long-term disabilities. The aging and increasingly obese world population, as well as the increasing incidence of diabetes, will continue to worsen the socioeconomic burden of chronic wounds and their complications [1-2].

A chronic wound is one that has been present for 30 or more days. Chronic wounds fail to proceed through the orderly and timely healing process that characterizes acute wound healing, leading to diminished anatomic and functional integrity of the injured site and increasing the likelihood of infection and further wound complications. More rapid healing of these wounds would result in decreased complications, decreased wound care requirements, and earlier return to daily activities, rapidly reducing the burden and cost of care [3].

Split-thickness skin grafting, or autografting, is currently the gold standard for the treatment of major traumatic and burn injury-related skin loss. Skin grafting provides regeneration of both the epidermis as well as underlying dermal elements and decreases wound contraction and extracellular matrix deposition compared with non-grafted full-thickness wounds [2]. However, because split-thickness skin grafting is limited by the availability of donor skin, the resultant donor sites are large, painful, and can themselves become chronic wounds, marked by delayed healing, hypertrophic scarring, and/or prolonged pain [4]. They are associated with immediate and significant pain due to the harvesting process, which exposes sensitive dermal pain receptors. In addition, potential development of pruritus, infection, dyschromia, delayed healing, and subsequent hypertrophic scarring can occur [5-6]. Additionally, since both the epidermis and dermis are captured, the graft retains components of the donor site, such as hair follicles and may not cosmetically match the surrounding skin [6].

Attempts to overcome these limitations have been made. Dr. Cicero Meek, a general practitioner in South Carolina, developed a method of tissue expansion to treat large body surface burns. This technology preceded meshing technology. Meek’s technology, developed in 1958, involved mechanical division of the skin graft, providing up to a 10-fold skin expansion, but pieces had to be placed dermal side down for success and, thus, was labor-intensive and time-consuming. Alternatively, cultured epithelial autografts (Epicel®, Vericel Corp, Cambridge MA) can provide an expansion ratio up to 1:1000, but the grafts are extremely fragile, lack a dermal component, and require extremely expensive techniques and facilities for development [5]. The Xpansion® Micro-autografting System (SteadMed Medical, Fort Worth, TX) uses split-thickness skin micrografts, which increase the expansion ratio to 1:100, is less labor intensive, and thus, can be performed in an outpatient setting with local anesthetic [5]. However, the system can only be used on small wounds, and donor site healing, while smaller than split-thickness skin grafting, is still associated with pain and scarring.

Epidermal blister grafting has traditionally been time-consuming, labor-intensive, and painful. Former harvesting techniques included using large-volume syringes to raise primarily epidermis-composed blisters, taking multiple procedures to complete, thereby, leading to an uncomfortable and time-consuming process. Additionally, inadequate handling of the graft could lead to tearing and improper orientation, resulting in graft failure [6]. The CelluTome™ Epidermal Harvesting System (Acelity Inc, San Antonio, TX was developed to help overcome these obstacles [4]. Per its accompanying literature, this device “is a harvesting tool that creates suction-epidermal blisters using a constant negative pressure of 400 to 500 mmHg at 37° to 41° C. The suction blisters are developed inside the disposable harvester, which consists of two stainless steel plates with an array of 1.75-mm holes and a cutter blade. More than 128 blisters can be created over an area of 25 cm2 of donor skin, which is then peeled away with a (transparent) dressing and applied over the recipient site” [5]. This technology offers a minimally invasive, relatively pain-free harvesting technique that creates minimal donor site damage and scarring, increased expansion ratios, and can be performed easily and relatively pain-free in the outpatient setting. The CelluTome™ technology makes epidermal grafting quality more consistent and the procedure economically and practically more feasible. As a result, the epidermal graft may have a role in reducing healing time in chronic and small acute wounds. Potentially, there could be a significant impact on the overall cost of chronic wound care. This case review series evaluates the outcomes of 13 recently treated patients with chronic wounds who underwent grafting of epidermal skin grafts using the CelluTome™ system. 

## Materials and methods

### Patient selection

Patients were selected based on the failure of their wounds to heal despite utilization of standard wound care treatments in the outpatient setting in our university-based community wound center. Standards of care at our wound center include optimization of moist wound-healing, drainage control measures, and usage of off-loading and compression in appropriate wounds. Patient characteristics are delineated in Table 1. We evaluated our patient selection criteria (type, size, and location of their wounds), comorbidities, patient age and sex, and, when applicable, time-to-wound-closure. In addition, by reviewing our billing documentation, we were able to estimate costs associated with wound care regimens prior to placement of the epidermal autograft.

**Table 1:** Patient Characteristics COPD: chronic obstructive pulmonary disease; DVT: deep vein thrombosis

Patient	Age	Sex	Pertinent Comorbidities
1	29	M	Smoker, Type I diabetes mellitus
2	36	F	Wheelchair bound, venous stasis, lymphedema,
3	52	M	Smoker, hepatitis C
4	36	M	Hypertension, asthma
5	72	M	Hypothyroidism, Type II diabetes mellitus, COPD, chronic kidney disease
6	60	M	Type II diabetes mellitus, asthma
7	37	M	Smoker, paraplegia/wheelchair bound
8	79	F	Venous stasis, hypertension, anemia
9	53	F	Smoker, history of DVT/ peroneal thrombosis
10	61	M	Coronary artery disease, Parkinson disease, obstructive sleep apnea
11	68	M	Hypertension, prostate cancer
12	62	M	Paraplegia/wheelchair bound, morbid obesity, Type II diabetes mellitus, peripheral neuropathy, lymphedema
13	69	M	Rectal cancer s/p neoadjuvant therapy and resection, ventral hernia s/p mesh placement, ileostomy, abdominal mucous fistula, COPD, hyperlipidemia

### Procedure: epidermal autograft harvesting and placement

The CelluTome™ Epidermal Harvesting System device was used to create epidermal micrografts for autograft placement to chronic wounds that had “stalled” in their healing trajectory, determined primarily by minimal change in size and/or appearance for two to four consecutive visits to the wound center. Verbal consent for the procedure was obtained prior to the procedure in all patients. Each donor site was prepped with 70% isopropyl alcohol after hair was clipped. The epidermal autograft harvester was adequately placed on the medial thigh in all but one patient, whose thigh was too large for the accompanied strap system, in whom the medial calf was used instead. Suction with a machine-setting negative pressure of 400 to 500 mmHg and warmth of 37° to 41° C were applied for an average of 44 minutes (range: 33 – 55 minutes), with a visually confirmed observation of sufficient development of epidermal blisters. A silicone-based, non-adherent dressing was placed over the blisters, confirming adherence. The CelluTome™ device was then activated, excising the blisters. Sufficient blisters were then noted on the dressing and were transferred by hand in the proper polarity to the patient’s surgically prepared (i.e. surgically debrided) recipient wound bed. All donor sites were dressed with a transparent adhesive dressing. All wound sites were dressed based on characteristics unique to each wound and the standard of care for that specific wound type. Follow-up was scheduled for approximately one week after application (six or seven days post-procedure).

### Wound care and measurement

The wound care regimen was patient and wound etiology-based. Standard wound care paradigms were used, i.e. maintenance of a moist wound environment, maintaining offloading when applicable, medical-grade compression utilization for venous leg ulcers, surgical debridement, and assessment and treatment for infection, if appropriate.

Wounds were measured in a standard fashion. The length was measured at its longest point, width at its widest, and depth at its deepest point. 

## Results

Thirteen wounds were treated. Five were leg ulcers with venous stasis or lymphedema, two were traumatic wounds, and three were diabetes-related wounds. Three wounds were at surgical sites, including one burn graft failure area, one neurosurgical dehiscence, and one abdominal wound with granulated mesh. Seven of the wounds were healed by three months after the epidermal graft placement, and another healed by four months after placement of the epidermal graft. Two were lost to follow-up, and three have not healed as of this time. One patient had two epidermal graft applications with healing approximately three months after the first application (Table 2). Specific patient information is delineated in Table 3.

**Table 2:** Outcome Summary

Outcome	Number of Patients
Healed at one month	4
Healed at two months	1
Healed at three months	1
Healed at four months	1
Not healed	3
Lost to follow-up	2

**Table 3:** Wound Characteristics and Progression

Patient, Etiology, and Location of Wound	Pre-CelluTome Appearance and Size	Wound Healing Progression; Days Post-graft; Size		Final Outcome
#1, Non–healing traumatic wound present for years due to DM; scalp	3.1 x 2.6 cm	7 days post-graft; 3.0 x 2.5 cm		Patient was lost to follow-up. Reevaluated approx 8 months later, local wound care ensued, and patient again lost to follow-up. Stated wound had healed and re-opened.
2, Ulcer secondary to venous stasis and lymphedema, present for years on the medial malleolus at the ankle (Calf donor).	3.0 x 2.6 cm	6 days post graft; 3.0 x 2.75 cm	29 days post graft; 1.8 x 1.8 cm with 0.8 x 0.8 cm open base	Wound closed at 85 days
3, Chronic dehisced surgical wound present for months following instrumentation s/p trauma, which was complicated by cervical osteomyelitis, failed STSG, and unsatisfactory negative-pressure wound therapy.	3.5 x 2 cm	6 days post graft; 3 x 3.5	41 days post graft; 4 x 3 mm opening with no depth	Wound closed at 57 days
4, Chronic wound of unknown origin present for months, treated at outside wound center initially, left lower extremity. Failed to progress with standard compressive therapy.	6 x 5 cm	6 days post graft; 5.5 x 3 cm (note epidermal blister grafts present on wound bed)	29 days post graft; 1 x 0.5 cm	Wound closed at 45 days
5, Diabetic foot ulcer, present for months complicated by osteomyelitis requiring fifth ray amputation which was treated with a wound vac for two months post-operatively; right fifth toe amputation site	5.5 x 1 cm	6 days post graft; 1 x 5 mm	13 days post graft; no open wound	55 days post graft; epithelium remains intact
6, Chronic diabetic foot ulcer complicated by osteomyelitis requiring amputation of first toe. Healing of amputation site was complicated by failed STSG and wound vac therapy; left first toe amputation site	6.5 x 4.5 cm	6 days post graft; 6 x 3.5 cm	27 days post graft; 6 x 2 cm	148 days post graft; 5 x 1.5 x 0.25 cm
7, Chronic lower extremity wound present for 15 months secondary to vascular disease; right calf	6 x 3.5 x 0.5 cm	7 days post graft; 6 x 3.5 x 0.5 cm	13 days post graft; 6 x 3.5 x 1 cm	Patient care transferred to other surgical service
8, Chronic venous stasis wound present for 23 months with failed Silver-based and Unna boot therapy; right medial malleolus	3 x 1.5 x 0.3 cm	6 days post graft; 3 x 1.7 cm	22 days post graft; 2.9 x 1.9 cm	
9, Chronic wound secondary to hematoma which was surgically debrided and treated with a wound vac; right thigh	3 x 2.1 cm	6 days post graft; 3 x 2.1 cm	13 days post graft; 2 x 1 cm	Wound closed at 29 days
10, Chronic wound present for months secondary to third degree burn with failed STSG; left lateral malleolus	5 x 1 cm	11 days post graft 3 x 1 cm	25 days post graft; no open wound	
11, Chronic lower extremity wound present for 3 years secondary to lymphedema and footwear trauma with previously failed Unna boot therapy; left foot near medial malleolus	4 x 2.6 cm	6 days post graft 4.5 x 2.5 cm	27 days post graft 3.5 x 2.5 cm	Repeat grafting was performed 34 days after the original graft due to slowed/stalled healing. Shown above is 21 days post second graft, measuring 1.9 x 1.7 cm
12, Chronic lower extremity wound present for >1 year secondary to diabetes; left medial foot	2 x 2.3 cm	6 days post graft; 2 x 2 cm	27 days post graft; 3 x 2 cm	34 days post graft; 3 x 1.5 cm Note: wound healed to 1.7 x 1.7 cm 20 days post graft, yet healing slowed thereafter
13, Large abdominal wound secondary to surgical complications; abdomen	14 x 5 cm	6 days post graft; 14 x 2 cm	20 days post graft; 13.2 x 5 cm	

## Discussion

**Figure 1:**
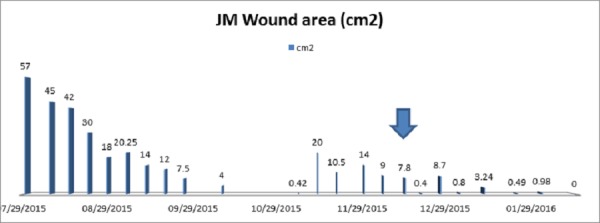
Representative Trend Line of Wound Healing in Patient 2 Lg arrow indicates placement of epidermal graft.

Using our billing and reimbursement data, we evaluated the healthcare costs associated with patient care, including clinic visits as well as procedural costs. Utilizing the Microsoft Excel (Microsoft Corp, Redmond WA) trend line function, we projected the timing of wound closure without the placement of an epidermal autograft. This led to an imperfect estimate, of course, as all of our patients were chosen to receive the placement of epidermal autograft specifically because of stalled wound healing. The trend line was based on the weekly wound measurements, which in some cases increased, stalled, or had reductions in size without closing (Figure 1). In our patients, wound closure was not expected in a reasonable timeframe. Thus, in spite of mathematic modeling describing wound healing over a period of 4+ weeks in the majority of cases due to the rate of prior wound size decrement, we clinically know this would likely not be the case in the majority, if not the totality of presented cases. However, it does give us a worthwhile idea of a potential timeline of subsequent wound care from which a cost of wound care may be estimated. Comparing costs of routine wound center-based procedures and dressings, we were able to hypothesize savings incurred by usage of this autograft technology for each patient. The comparatively rapid closure of the open wound(s) post-epidermal autograft placement potentially reduces healthcare costs based on charges at an average of $1,153 per patient (Table 4).

 

**Table 4:** Potential Savings Due to Reduced Healing Time NA: not applicable; TCC: total contact cast

Pt #	Saved clinic charges
1	NA, lost to follow-up
2	$2,148 (6 wk Unna boot)
3	$852 (4 clinic visits)
4	$1,252 (4 week Unna boots)
5	$2,100 (4 weeks TCC)
6	NA, still open wound
7	NA, lost to follow-up
8	$1,252 (4 weeks Unna boots)
9	$768 (4 weeks vac dressing)
10	$852 (4 weeks clinic visits)
11	NA, still following
12	NA, still following
13	NA, still following

Specifically, estimated costs associated with wound center-based care based on billing information are as follows:

- Clinic f/u fee, level 3 visit: $122/visit

- Clinic facility fee: $91

- Professional fee for Unna boot: $100/application

- Professional fee vac change: $70/procedure

- Unna boot facility fee: $136/application

- Average total contact cast (TCC) application professional fee: $434/application

- Average charge of placement of epidermal autograft in wound center setting: $1,827

- Average reimbursement for placement of epidermal autograft: $530

The CelluTome™ Epidermal Harvesting System (Acelity Inc, San Antonio, TX) device has costs associated with its usage as well. The cost of one harvester is approximately $350 for our wound center. The harvesters are non-reusable; any repeat applications (performed on one patient at the time of this submission) incur a new harvester being used with a resultant duplication of the costs. Procedural charges are significant but, in all but two cases, have yielded significant payments to the wound center, averaging approximately $650 for the nine patients on whom billing data and insurance payment data is currently available, including two pending payments. Regardless of payment information, however, the prospect of earlier wound closure, earlier cessation of wound care and its resultant costs, and, most importantly, even in today’s financially-sensitive environment, earlier return of the patient to their normal life without the physical, financial, and emotional burden, of a chronic wound cannot be overestimated. Even if this procedure was entirely cost-neutral, which it has not been, the benefit of healing a recalcitrant wound in a wound healing center cannot be overestimated. Patient satisfaction improves remarkably in healed patients as does physician satisfaction, nursing satisfaction, and family satisfaction. 

## Conclusions

The CelluTome™ Epidermal Harvesting System (Acelity Inc, San Antonio, TX) device markedly accelerated healing in eight of the 13 patients we treated at the time of the writing of this article. Unfortunately, two patients were lost to follow-up. Nonetheless, by accelerating wound healing in our patient population, all costs associated with subsequent wound care seem to have decreased to a dramatic degree and wound center finances have improved. Even more dramatically, the healing our patients experienced was gratifying and honestly surprising in certain patients (specifically, Patients 2 and 8, whose wounds were treated for years prior to seeking care with our wound specialists). Even in accounting for the expense of the harvester and the professional fees associated with the procedure, significant cost savings were noted with the usage of the CelluTome™ system in those patients who healed, i.e. 8/13 (62%) of the patients whom we treated. The procedure was well tolerated by all of the patients, and thus far, in our patients who healed, no wound recurrence has been observed since starting to use this technology and grafting technique over the past year. Given these improved outcomes, cost-savings, and a better financial outlook for the wound center, utilization of the CelluTome™ Epidermal Harvesting System is proving itself to be in the “win-win” category of wound care treatments with little-to-no downside, markedly improved healing rates, and subsequent improvement in patient, caregiver, and staff satisfaction.
